# Automated Data Quality Assessment of Marine Sensors

**DOI:** 10.3390/s111009589

**Published:** 2011-10-11

**Authors:** Greg P. Timms, Paulo A. de Souza, Leon Reznik, Daniel V. Smith

**Affiliations:** 1 Tasmanian Information and Communication Technologies Centre, Commonwealth Scientific and Industrial Research Organisation (CSIRO), Hobart, TAS 7001, Australia; E-Mails: paulo.desouza@csiro.au (P.A.S.); daniel.v.smith@csiro.au (D.V.S.); 2 Department of Computer Science, Rochester Institute of Technology, Rochester, NY 14623-5608, USA; E-Mail: lr@cs.rit.edu

**Keywords:** sensors, measurement results, quality, fuzzy logic

## Abstract

The automated collection of data (e.g., through sensor networks) has led to a massive increase in the quantity of environmental and other data available. The sheer quantity of data and growing need for real-time ingestion of sensor data (e.g., alerts and forecasts from physical models) means that automated Quality Assurance/Quality Control (QA/QC) is necessary to ensure that the data collected is fit for purpose. Current automated QA/QC approaches provide assessments based upon hard classifications of the gathered data; often as a binary decision of good or bad data that fails to quantify our confidence in the data for use in different applications. We propose a novel framework for automated data quality assessments that uses Fuzzy Logic to provide a continuous scale of data quality. This continuous quality scale is then used to compute error bars upon the data, which quantify the data uncertainty and provide a more meaningful measure of the data’s fitness for purpose in a particular application compared with hard quality classifications. The design principles of the framework are presented and enable both data statistics and expert knowledge to be incorporated into the uncertainty assessment. We have implemented and tested the framework upon a real time platform of temperature and conductivity sensors that have been deployed to monitor the Derwent Estuary in Hobart, Australia. Results indicate that the error bars generated from the Fuzzy QA/QC implementation are in good agreement with the error bars manually encoded by a domain expert.

## 1. Introduction

Computerization of modern science and technology has raised many concerns about the methods traditionally used to ensure the integrity and utility of data ([1], and references therein). It is particularly important that sensor deployments and their associated information systems follow scientific fundamentals to ensure the integrity and validity of the data collected and presented. These principles were recently formalized by the U.S. National Academy of Sciences Committee on Ensuring the Utility and Integrity of Research Data in the Digital Age in their report [2].

Addressing the report recommendations and the basic principles of scientific research, this paper develops a methodology and implementation procedures for automatically assessing the quality of research data gathered from sensors. In particular, it concentrates on calculating and presenting data quality metrics along with the research data themselves, crucial as modern measurement theory and practice assumes getting a measurement result along with some characteristics of its uncertainty [3]. Since the early 1990s and in particular since the publication of the ISO Guide [4], there has been an increasing recognition that the uncertainty of a measurement is no less critical than the value of the measurement result itself. Uncertainty may have a number of different components. Some of these may be evaluated by statistical methods but others may require expert estimates, reasoning and judgment ([4], and references therein). This paper combines both approaches. It derives the total uncertainty characteristics from statistical processing of the available data and includes a contribution from domain expert judgment.

The paper’s goal is twofold:

- to describe, for the first time, an automated method that uses a prototype fuzzy rules expert system to generate error bars. This will provide a more meaningful measure of uncertainty than a hard classification using data flags;
- to demonstrate this approach’s success through its application to marine water monitoring.

## 2. Research Data Quality and its Evaluation through Measurement Uncertainty

Standards, e.g., *International Guide to the Expression of Uncertainty in Measurement* [4] (or GUM as it is now often called) and its US equivalent ANSI/NCSL Z540-2-1997 *US Guide to the Expression of Uncertainty in Measurement* [5] request the provision of a quantitative indication of the quality of the measurement result along with the result itself, so those who use the measured data can assess its reliability. The GUM standard’s approach groups the components of uncertainty in the result of a measurement into two categories according to the way in which their numerical value is estimated: those which are evaluated by statistical methods are classified as “Type A”, and those which are evaluated by other means are classified as “Type B”. A Type B evaluation of standard uncertainty is usually based on scientific judgment using all of the relevant information available, which may include:

- previous measurement data;
- experience with, or general knowledge of, the behavior and property of relevant materials and instruments;
- manufacturer’s specifications;
- data provided in calibration and other reports, and uncertainties assigned to reference data taken from handbooks.

As real-time sensor platforms become the norm in environmental sensing, there is a need to develop automated procedures to incorporate scientific judgments of the streamed data in the evaluation of the measurement uncertainty and associated data quality. As the judgments are often based on experts’ opinions and estimates, the data quality assurance systems could be designed within an expert system framework that uses fuzzy rules to characterize the properties and sources of the judgment.

Fuzzy systems have been used in applications where the solution is highly dependent on human experience; because of either imprecise information being available or the empirical nature of the problem (e.g., [6], and references therein). Using a fuzzy system, it is possible to encode linguistic rules and heuristics, reducing the solution time since the expert’s knowledge can be built in directly. In addition, its qualitative representation form makes fuzzy interpretations of data very natural and an intuitively plausible way to formulate and solve several problems. Qualitative aspects can be implemented and can also be updated making this system useful to solve problems that are very difficult or impossible to solve analytically.

Fuzzy heuristics can be applied to model a measurement result and its uncertainty [7,8], classifying the existing models of measurement uncertainty into the following groups:

1. Statistical model (standard model of uncertainty): the measuring function *f* is a random function and the measurement results *Xi* are real numbers. NB In this model the type B component of inexactness may not be well represented.
2. Fuzzy set model: the measuring function is fuzzy, and the space Y of measured results are the fuzzy intervals *J_Y_* characterized by the membership function *μ*(*x*) = μ_J(x)_ (x). In this model:
  - The result of a single measurement is a fuzzy interval; the uncertainty of this measurement is described by a membership function;
  - The mathematical operations performed on the measurement results are operations on fuzzy intervals. Arithmetic operations on fuzzy intervals could be defined in a variety of different ways.
3. Random-fuzzy model: the measuring function *f* is a random function, and the space Y of measurement results is the set of fuzzy intervals *J.* In this model, the results of measurements are random fuzzy intervals. It is possible to define the extended uncertainty for a given significance level using the fuzzy α-cut technique [9].

Over the last few years, new applications demonstrating more complicated schemes for using fuzzy formalized expert information in measurement procedures have been published. Mahajan *et al*. [10] describe an intelligent fusion measurement system in which the measurement data from different types of sensors with various resolutions are fused together based upon a measure of their confidence. This confidence was derived from information not commonly used in data fusion, such as operating temperature, frequency range and fatigue cycles, which are fed as additional inputs to a fuzzy inference system (FIS) with predefined membership functions. The outputs of the FIS are weights that are assigned to the different sensor measurement data reflecting the confidence in the sensor’s behavior and performance. In [8] the fuzzy and interval analysis models of a two-dimensional navigation map and rough estimates of a robot position are applied to improve robot guidance and navigation. Mauris *et al*. [11] aim at reproducing the linguistic evaluation of comfort perception provided by a human through aggregation of relevant physical measurements such as temperature, humidity, and luminosity.

These systems all combine different aspects to achieve a quick assessment of interest and our work shares this same objective by combining qualitative and quantitative assessments of sensor measurements to produce an automated data quality assurance system for marine sensor deployments.

## 3. Automated Data Quality Control for Marine Sensors

Despite a lack of data quality assessment in deployed near real-time marine sensors, there have been some notable exceptions. For example, the Argo float project has deployed over 3,000 profiling floats with satellite communications capabilities throughout the world’s oceans, and could be regarded as one of the largest sensor networks in operation worldwide. A key aspect of the project is both automated and manual QC of the data collected by the floats [12]. In recent years, a series of workshops have also been held in the USA to address the quality assurance of real-time marine data. Whilst recommending quality assurance procedures to improve the raw data quality, the participants also recognized the importance of automated QC including range and gradient checks on data as well as routines to remove data spikes and to take into account the output of nearby sensors [13]. Koziana *et al.* [14] describe their approach which also makes use of range and gradient checks on data. In all these cases, the outcome of the QC process is a data quality flag associated with each measurement; information on which quality checks have been passed or failed is often also made available.

Whilst the provision of a data quality assessment is a significant improvement over its absence, it still leaves a challenge for the data user (who may not be a domain expert) in determining whether the data is fit for their purpose. For example, a swimmer or surfer looking at real-time water temperature data from local beaches may be content with uncertainties of 0.5 °C (0.9 °F) in the quoted data. But, can they rely on data flagged as “probably good”? In practice, the swimmer or surfer would need to consult a domain expert (impractical in most cases) to know whether data flagged “probably good” is fit for their purpose. In contrast, the work reported here seeks to quantify the uncertainty of individual measurements, effectively encoding domain expertise in the stored data themselves.

In related work, Faradjian *et al.* [15] proposed abstract data types (ADT) and data structures for “indexing” noisy sensors. Data from the sensors are represented as probability density functions to take into account the uncertainty associated with each data point. However they do not generate the uncertainties but assume the measurement mean and standard deviation as inputs for their data types and algebra.

A number of theoretical papers in the computer science literature have dealt with the challenge of dealing with incomplete and imprecise information. Parsons [16] provides a good review of this work in the research fields of databases and artificial intelligence. Of most relevance is the work of Umano [17] who combines the use of possibility distributions to model uncertainty in the value of an attribute and fuzzy degrees of membership to model the degree of association between values.

The work of Doong *et al.* [18] is the most closely related to the work presented here. They point out that “Data quality control is based on both objective criteria and human experience” and outline an automated data QC procedure for their coastal ocean monitoring network. A key difference in our approach is the use of fuzzy logic to result in a continuum in the data quality assessment and hence the ability to automatically assign error bars to data. This capability is crucial for data re-purposing, meaningful comparisons with models, prediction of sensor failure and scheduling of network maintenance.

Parsons concludes that “it would be both interesting and useful to study the imperfections in data that real systems come up against from the perspective of actually building such a real system rather than studying the problems of imperfect data in a theoretical vacuum.” Bettencourt *et al.* [19] demonstrate this maxim with a practical anomaly detection scheme for wireless networks and, in the present paper; we develop and apply our approach to data from the Tasmanian Marine Analysis Network.

## 4. Case Study: The Tasmanian Marine Analysis Network

**Figure 1.**
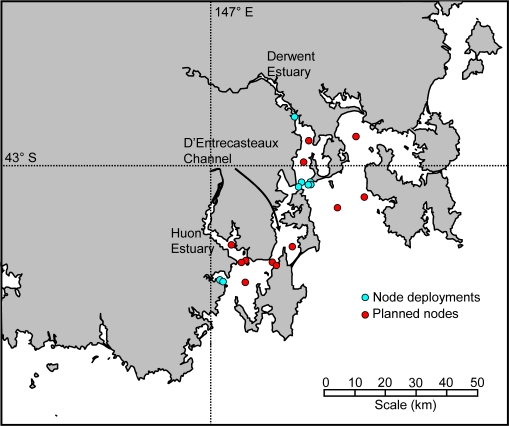
Location of node deployments and planned nodes in the TasMAN network.

The Tasmanian Marine Analysis Network (TasMAN) [20] has been developed and deployed to help manage the multiple uses of the estuaries and coastal regions of southern Tasmania, Australia (Figure 1). These regions are used by industry, shipping, aquaculture and tourism operators along with commercial and recreational boaters and fishers. The network is designed to collect real-time data that will help monitor the health of the estuaries, as well as to provide warnings to industry and the general public.

South-eastern Tasmania is home to two estuaries with very different histories. The Derwent estuary flows through the Tasmanian capital of Hobart. While the water quality is now improving, it has been greatly impacted in the past by urban stormwater and sewerage, industry (including a zinc smelter and paper mill), dense agricultural activity in the catchment, shipping, and commercial and recreational boating and fishing. In recent years, regulations and programs have led to a dramatic increase in water quality; however the riverbed sediment remains amongst the most polluted in the developed world [21]. The neighbouring Huon estuary has its headwaters in a World Heritage Area that makes up the southwest of the state. This region is largely untouched, mainly because of its remoteness and ruggedness. While there is some agriculture in the lower reaches of the catchment, and some aquaculture (salmon farming) near the mouth of the estuary, it is in a near pristine condition.

The TasMAN network consists of fixed and mobile nodes and uses mobile (3G) communications to deliver real-time data from the two estuaries, and the D’Entrecasteaux Channel which joins them, back to the CSIRO site in Hobart where they are integrated with models to produce nowcasts and forecasts of river conditions. It is also a demonstration infrastructure where sensor data are exposed for different applications and through different platforms. In these applications, data quality should be assured.

## 4. Data Quality Control in TasMAN Using Fuzzy Rules

### 4.1. Sensor QAQC Inputs

A fuzzy rules-based system was implemented to assess the data quality at the sensor level. The system includes provisions for both Type A (rate of change of output values, cumulative rate of change of output values and node differences) and Type B (time since last calibration and time since last maintenance) uncertainty parameters. The rules’ inputs are as follows:

#### 4.1.1. Time since Last Calibration

The output of a sensor drifts over time with a given rate. These drifts are generally related to the quality of a sensor and, hence, sensors require regular calibration. If a sensor is not calibrated according to the schedule outlined by the manufacturer, confidence in data obtained from the sensor is reduced.

#### 4.1.2. Time since Last Maintenance

Sensors will be affected, to a greater or lesser degree, by the environment in which they are deployed. Sensors exposed to excessive heat or dust for long periods of time may degrade in performance. This parameter is particularly important in a marine environment because of the presence of high salt concentrations and biofouling (algal growth, shellfish attachment, *etc.*).

**Figure 2.**
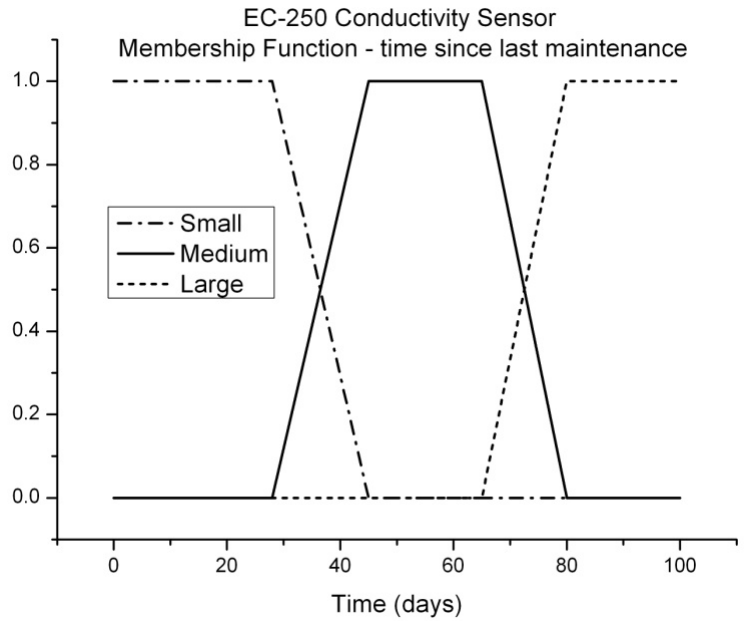
Membership function for the parameter, time since last maintenance, for a number of sensors used in the TasMAN network.

An example of this membership function, for one of our temperature sensors (EC-250) and the Seabird37 conductivity, temperature and pressure sensors, is shown in Figure 2. Here, S (small), M (medium) and L (large) refer to the error introduced in the sensor output by this parameter, time since last maintenance.

**Figure 3.**
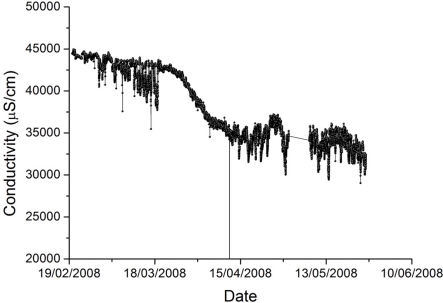
Conductivity data, at a depth of one meter at the CSIRO Wharf, showing an example of a plateau followed by a drop in sensor data quality.

The shape chosen for the membership functions reflects the observation that measurement quality from our marine sensors appears to plateau at various stages as it degrades. Figure 3 shows a conductivity time series where this plateau effect is prominent. The reason for the plateau behavior is not clear but may be a result of the growth of different organisms on the sensor (e.g., initially algae, followed later by shellfish).

#### 4.1.3. Rate of Change of Output Values

The rate of change of the sensor values (*RoC*(*t*) = *x*(*t*) − *x*(*t−1*)) was used as an input. Given the large-scale physical processes under study, rapid changes in sensor output are typically due to faulty sensors and not to sudden changes in the environment (e.g., given the high specific heat capacity of water and the large volume present, it is unlikely that water temperature in the estuary would rise by 1 °C over 5 min). Instead, typical timescales for changes in physical parameters are hours or days.

#### 4.1.4. Node Differences QA/QC

Node differences QA/QC compares the observations of individual sensors (of the same type) co-located within the same node and identifies any large discrepancies. The output from this process is used to modify the overall data quality assessment at the sensor level. The modification could be either downwards or upwards. For example, if all sensors in the node have recorded a decrease of 2 °C in water temperature in the past 30 min, then it may indicate an event at this location and we can have more confidence in the data from the individual sensors. However, if only one sensor records this decrease, it is suggestive of a fault with that sensor.

Where there are only two sensor locations on a node, the percentage difference between the sensor readings could be used as the metric for Node QA/QC. In cases with more than two sensors it would be preferable to compare the sensor reading with the mean of other sensors on the same node. Table 1 shows the characteristics of a membership function for a case where there are two sensors of the same type at the same node location.

This approach should be modified depending on the system under study. In the results presented below, the water body was well-mixed and data at the two sensors would be expected to be similar, despite their different depths. However, layering typically occurs in estuaries with warmer, less brackish water near the surface and cooler, more brackish water at depth. For these cases, it may be preferable to calculate water density from sensor readings at each depth and use these to check that the measured water column was hydrostatically stable. Contrastingly, a statistical model of the relationship between the two sensors in the stratified water column could be utilized as a quality metric. The model could be developed from training data and the metric could represent the difference between the model prediction and actual different between the two sensors.

#### 4.1.5. Cumulative Rate of Change of Output Values

For the case of the “rate of change” parameter in Equation (1), a further membership function was needed. A sudden spike in a parameter (e.g., temperature) may be followed by a gradual decline back to its previous level. In this instance, the automated QA/QC system would detect the spike and associate a large error with it, but would not associate a large error with the subsequent readings (as the rate of change was small on each occasion). To address this issue, a *cumulative rate of change* function was introduced to retain some memory of previous rates of change. For the first measurement, the cumulative rate of change (cRoC) membership function was equal to the RoC function. For a following data point, at timestep *t*, it was calculated via:

(1) $$\begin{array}{l} \text{if}\;({\text{RoC}}_{\text{small}}\;(t)+0.5\times {\text{RoC}}_{\text{medium}}\;(t))<({\text{cRoC}}_{\text{small}}\;(t-1)+0.5\times {\text{cRoC}}_{\text{medium}}\;(t-1))\;\text{then}\\ \;\;{\text{cRoC}}_{i}\;(t)={\text{RoC}}_{i}\;(t)\\ \text{else}\\ \;\;{\text{cRoC}}_{i}\;(t)=(1-k)\times {\text{RoC}}_{i}\;(t)+k\times {\text{cRoC}}_{i}\;(t-1)\\ i=\text{small},\;\text{medium},\;\text{large} \end{array}$$

where *i* is the index of the fuzzy set parameters and *k* is the smoothing time constant. A large value of the constant *k* results in a longer time for the return of confidence in sensor data following a spike. The first case in Equation (1) ensures that the cumulative rate of change function is able to quickly respond to degradations in sensor quality, while the second case ensures that it takes a longer time for confidence to return in the measurements from that sensor once the rate of change of the parameter decreases. Table 1 shows the characteristics of the membership functions for sources of uncertainty in the TasMAN network.

### 4.2. Combination of Fuzzy Sets for Quality Assessment

For a given measurement, “small”, “medium” and “large” uncertainty values were assigned for each of the parameters in Table 1. Empirically determined weightings were provided to the different parameters and they were combined according to:

(2) $$\begin{array}{c} {\text{Overall}}_{i}=0.2\times {\text{TM}}_{i}+0.1\times {\text{TC}}_{i}+0.25\times {\text{RoC}}_{i}+0.25\times {\text{cRoC}}_{i}+0.2\times {\text{Ndiff}}_{i}\\ i=\text{small},\;\text{medium},\;\text{large} \end{array}$$

where TM is the “time since last maintenance” membership function, TC is the “time since last calibration” membership function and Ndiff is the “node difference” membership function.

Once combined, the overall “small” value was referred to as the QC green fraction, the overall “medium” value as the QC yellow fraction and the overall “large” value as the QC red fraction. Finally the data was compared against thresholds (Table 2) based on the range of historical measurements in the region. Where data fell outside the expected range, the small and medium error fractions were overwritten with 0 and the large error fraction overwritten with 1. It should be emphasized that the data samples are not being altered according to this assessment, quality assessments of the samples are only being provided.

### 4.3. Using Fuzzy Assessments for Generating Errors Bars

The defuzzification process used to provide an overall estimate of data quality assigned uncertainties to the green, yellow and red components of the overall membership function. By combining these uncertainties, an overall quantitative assessment of uncertainty for each measurement was generated via Equation (3):

(3) $$\begin{array}{c} \text{Error}=\Sigma \;\alpha_{I}\times {\text{Overall}}_{i}\\ i=\text{small},\;\text{medium},\;\text{large} \end{array}$$

α_small_ was assigned a value equal to the sensor accuracy quoted by the manufacturer; the other coefficients were multiples of α_small_ that were chosen empirically by trial and error using early 2008 data as a training set. Table 3 lists the values of α obtained.

## 5. Results and Discussion

The system was initially applied to data collected at the CSIRO Wharf in Hobart between 25 August and 5 November 2010. This node was composed of two EC-250 temperature and conductivity sensors fixed to the wharf, one at a depth of 1.0 m below chart datum and the other at a depth of 9.5 m below chart datum (chart datum was at the level of the lowest possible astronomical low tide). The sensors were field calibrated in early September 2010, following their deployment on 25 August 2010.

The automated data QA/QC procedure described in Section IV was applied to this dataset with the constant *k* assigned a value of 0.8. A subset of this data was also processed manually to generate error bars for comparison with the automated system. The subset was picked to represent data collected throughout the deployment and included all data collected on 26 to 28 August, 12 to 13 September, 26 to 27 September, 10 to 11 October and 25 to 26 October; a total of 2,672 datapoints from each sensor.

**Figure 4.**
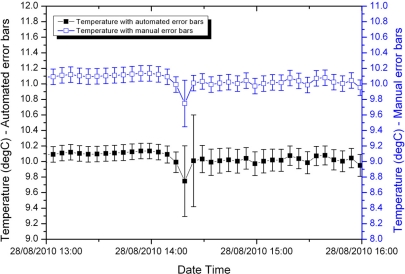
Shallow water temperature with manually calculated and automatically generated error bars for a period early in the sensor deployment.

**Figure 5.**
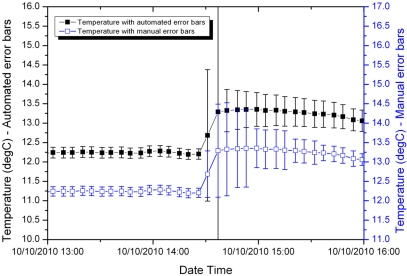
Shallow water temperature with manually calculated and automatically generated error bars for a period late in the sensor deployment.

Figures 4 and 5 compare the manually calculated error bars and the error bars generated automatically for two samples from the shallow temperature dataset, one early in the deployment and the other later in the deployment.

Prior to the temperature spike in Figure 4, the manually calculated and automatically generated error bars are close to identical. This is because this early in the deployment the main contribution to error is the accuracy of the sensor itself. The fuzzy set system indicated an overall small uncertainty; through Equation (3) this results in a calculated error bar equal to the sensor accuracy. During and immediately following the spike, the fuzzy set system slightly overestimates the error associated with the measurement. This is a conservative approach and can be corrected later when the data is inspected by a domain expert.

Later in the deployment, the measurement errors are larger (note the greater range of temperature on the y-axes in Figure 5). In this case, the fuzzy set system performs well prior to the jump in temperature. During the jump it conservatively overestimates the error. Immediately following the jump it slightly underestimates the error, but is still within a factor of two of the manually calculated error bars.

The automatically generated error bars were expressed as a percentage of the manually-determined error bars over the 2,672 datapoints for each of the four sensors. Table 3 presents the distribution of these calculated percentages.

The fuzzy system is much more successful when applied to the temperature sensors than to the conductivity sensors. In the case of the temperature sensors, the automatically generated error bars are within 50% of the manually determined error bars for approximately 80% of the time, compared with approximately 37% in the case of the conductivity sensors. The fuzzy system is also more successful at estimating error bars for the deeper sensors than for the shallower sensors. The deeper sensors are less susceptible to biofouling than those near the surface, and biofouling has a much more significant effect on the conductivity sensors than on the temperature sensors, so the distributions shown in Table 3 suggest that further work needs to be done in order to accurately account for biofouling effects in the automated process.

The fuzzy system has a tendency to overestimate the error bars. This is conservative as, in these cases, the true value is still likely to lie within the error bars; however the range indicated by the error bars is larger than is necessary.

Overall the novel approach detailed in this paper represents a viable approach to automatically generating error bars, producing error bars within 50% of the manually determined value approximately 80% of the time, and producing error bars within a factor of three approximately 99% of the time, in the case of the temperature sensors which are less susceptible to biofouling.

## 6. Conclusions

The automated collection of data streamed through sensor deployments has led to a massive increase in the quantity of environmental and other data available. The real-time requirement of streamed data and its sheer quantity means that automated data QC is necessary to ensure that the data collected is fit for purpose.

We have designed and implemented a novel prototype expert system for automated data quality assessment, which incorporates not only data statistics, but also the expert knowledge of domain specialists. The framework has been successfully applied for evaluating and presenting the quality of data collected through a marine sensor network in South-Eastern Tasmania, Australia. Overall, in the case of the temperature sensors which are less susceptible to biofouling, the approach detailed in this paper produced error bars within 50% of the manually determined value approximately 80% of the time, and produced error bars within a factor of three approximately 99% of the time. The approach outlined here is able to assess data quality of large datasets in a timely manner and it is being applied to real-time data streaming from the TasMAN network.

## Figures and Tables

**Table 1.** Membership functions for the parameters contributing to data quality.

**Parameter**	**Small**	**Small → Medium**	**Medium**	**Medium → Large**	**Large**
Rate of change of temperature (°C/min)	<0.03	0.03 to 0.05	0.05 to 0.07	0.07 to 0.11	>0.11
Rate of change of conductivity (μS/min)	<50	50 to 100	100 to 150	150 to 250	>250
Time since last calibration (days)	<360	360 to 540	n/a	540 to 720	>720
Time since last maintenance (days): all temperature sensors, SBE37 conductivity and pressure sensors	<60	60 to 120	120 to 150	150 to 210	>210
Time since last maintenance (days): EC-250 conductivity sensor	<28	28 to 45	45 to 65	65 to 80	>80
Percentage difference between sensor readings	<1.5%	1.5% to 3.5%	3.5% to 6.5%	6.5% to 10.5%	>10.5%

**Table 2.** Thresholds applied to physical parameters of temperature and conductivity.

**Parameter**	**Minimum value accepted**	**Maximum value accepted**
Temperature (°C)	5	25
Conductivity (μS/cm)	25,000	60,000

**Table 3.** Empirically determined coefficients used in Equation (3).

**Parameter**	**Small**	**Medium**	**Large**
Temperature (°C)	0.1	0.3	1.2
Conductivity (μS/cm)	200	1,000	8,000

**Table 3.** Ratio of automatically generated to manually determined error bars (expressed as a percentage) for eleven days chosen to be representative of the sensor deployment period.

**Automatically generated error bar/Manually determined error bar**	**Temperature (−1 m)**	**Temperature (−9.5 m)**	**Conductivity (−1 m)**	**Conductivity (−9.5 m)**
Above 300%	13 (0.5%)	1 (0.0%)	547 (20.5%)	537 (20.1%)
Between 200 and 300%	166 (6.2%)	339 (12.7%)	268 (10.0%)	365 (13.7%)
Between 150 and 200%	119 (4.5%)	84 (3.1%)	336 (12.6%)	342 (12.8%)
Between 66.7 and 150%	2,131 (79.8%)	2,224 (83.2%)	980 (36.6%)	993 (37.2%)
Between 50 and 66.7%	108 (4.0%)	9 (0.3%)	99 (3.7%)	138 (5.2%)
Between 33.3 and 50%	92 (3.4%)	7 (0.3%)	177 (6.6%)	140 (5.2%)
Below 33.3%	43 (1.6%)	8 (0.3%)	265 (9.9%)	157 (5.9%)
